# Day and night glucose control using a hybrid closed loop system for the management of type 1 diabetes

**DOI:** 10.1186/1687-9856-2015-S1-P26

**Published:** 2015-04-28

**Authors:** Martin de Bock, Anirban Roy, Julie Dart, Barry Keenan, Elizabeth Davis, Timothy Jones

**Affiliations:** 1 Princess Margaret Hospital for Children, Perth, WA, Australia; 2 Telethon Kids Institute, The University of Western Australia, Perth, WA, Australia; 3 School of Paediatrics and Child Health, The University of Western Australia, Perth, WA, Australia; 4 Medtronic Minimed, Northridge, California, USA

## 

Achieving tight glycemic control for the management of type 1 diabetes is often associated with a heavy burden of care, and frequent hypoglycemia. Closed loop insulin delivery, or an “artificial pancreas”, represents a new technological frontier for the management of type 1 diabetes which aims to overcome these difficulties. Rapid advancement and improvement in the components of closed loop systems (continuous glucose monitors, insulin pumps, and mathematical algorithms) has been translated into numerous published reports demonstrating effective glucose control in hospital studies, camps, hotels, and most recently – in home studies. Most studies to date have focused on overnight control due to the difficulties in managing glucose excursions from carbohydrate intake and exercise. We investigated the capability of the Medtronic Hybrid Closed Loop (HCL) System in managing glucose levels during both day and night. The Medtronic HCL system consists of a Medtronic MiniMed insulin pump, Medtronic MiniMed Enlite II glucose sensor, Medtronic MiniMed Minilink REAL time sensor, Medtronic MiniMed Translator, and an Android mobile device with the HCL algorithm (proportional integrative derivate minus insulin feedback and additional safety parameters) software application installed. When using the HCL, meal boluses are delivered manually using the Android mobile device. All basal insulin delivery is controlled by the algorithm. We present in-clinic pilot data from 3 individuals with type 1 diabetes, incorporating 140 hours of closed loop management, over 5 days and nights. A free living environment was simulated, with free access to food, and exercise encouraged. For the three participants, percent of time spent in target glucose range (4 – 10mmol/L) was 81%, 62% and 73% respectively. Including all data, the mean glucose was 8.74mmol/L, which corresponds to an HbA1c of approximately 7%. There were no hypoglycemic or adverse events. We conclude that the Medtronic HCL system is potentially effective and safe for the management of type 1 diabetes. Outpatient studies, with comparisons to sensor augmented pump therapy with low glucose suspend in a randomized cross-over trial are ongoing.

